# Supplementary material for: Sequence context and methylation interact to shape germline mutation rate variation at CpG sites
Source: PLoS Genet. 2026 Jun 1;22(6):e1011957. doi: 10.1371/journal.pgen.1011957 (PMC13245866; doi:10.1371/journal.pgen.1011957)
Supplement: Attachment — Submitted filename: Response_to_reviewers_v2.pdf [file pgen.1011957.s020.pdf]

Response to reviewers

We thank the editor and reviewers for their thoughtful comments. We have revised the manuscript to address their suggestions through three major analytical updates: (1) including singletons in all our analysis of polymorphisms, (2) validating our results by comparing with existing models as well as independent mutation data (de novo mutations), and (3) performing additional analyses on the effects of recombination, hydroxy-methylation, and replication on context-dependent effects. We have also reframed our study as an effort to understand the biology behind CpG mutation rate variation by focusing on the distinct mutation properties of unmethylated and methylated cytosines, and refrain from presenting our work as a new or better model for estimating site-level mutation rates. We now also report the estimated scaled mutation rates for each 4-mer and 6-mer sequence context in all the species studied in a supplementary table (S1 Table). Please see our point-to-point response below.

While the manuscript was under review, we evaluated the effects of different CpG filtering and SNP polarization methods and found that the rank-order of contexts in all three primates remained consistent. However, we found that using an “intersected genome”–extracting CpG sites from the reference genome that are also represented in the ancestral reference genome–and using the major allele for ancestral state polarization yielded the best results in terms of variance explained (see S4 Table). Therefore, we have revised all the analyses in the manuscript accordingly for all primate species, notably, all results remain qualitatively robust to the choices of CpG definition and SNP polarization method.

In addition, we re-did the chimpanzee analysis with a new, larger polymorphism (n=196) dataset published recently [1] that was not available during our original analysis. The new analysis yielded qualitatively similar results regarding independent effects of adjacent nucleotides, rank-order of contexts, marginal effects of flanking bases, and mutation asymmetry at the same CpG. However, the larger dataset resulted in much better concordance in context effects between chimpanzee and either rhesus or human at both methylated and unmethylated CpGs, suggesting potential technical artifacts in the previous, smaller chimpanzee polymorphism dataset (n=34). While our results now suggest conserved context effects at unmethylated sites across all three primates, we still observe that context effects have diverged at methylated sites in the chimpanzee lineage. We have updated the text and figures to update this new finding.

With these updates, we believe our revised manuscript is technically more robust and clearer regarding the motivations and significance.

Editor's Comments

Fig 1A is too dense to be of much use. Connecting line segments are a bit misleading when bins are modelled independently, they can be a visual aid but here the overlapping undermines that. I can't at the moment recommend a better way to present the data - it will require some exploration.

Table 1: since you discuss number of parameters in each model perhaps this should be included in the table. Your own discussion in the table caption illustrates that AIC is not much use when sample sizes are large, because model complexity is insufficiantly penalised. BIC may be more useful.

We thank the editors for these suggestions. We have modified Fig. 1A to show the polymorphism rate across all sites instead of 16 separate curves for each 4mer context. We agree this streamlines the visualization and ensures that the shape of the curve–which is our primary focus–is not obscured by overlapping data. However, because the variation in polymorphism curves across contexts provides an intuitive illustration of context effects conditional on methylation, we have relocated the original version to S1 Figure for interested readers.

As suggested, we have also updated the table (now Table 2) to include the BIC values to provide a more robust comparison of model fit.

Reviewer #1:

In this study, the authors report that germline mutation rates at CpG dinucleotides are affected by the broader context in a manner dependent on the methylation of the C. This report of statistical interaction of the DNA methylation and the oligonucleotide mutation risk is to my knowledge novel. The study is however not fully developed in terms of validating the effect in independent data, comparing against baselines, and showing at least some mechanistic implications of the result.

1- Could they show predictive value on truly independent mutation data, ideally de novo germline mutations (trios) rather than polymorphisms; alternatively or in addition, somatic mutation analysis would certainly not be uninteresting either.
This should support the claim that their methylation * context model improves prediction beyond existing sequence-context-only models and beyond methylation alone.

We thank the reviewer for this suggestion. We now include a comparison of our mutation rate estimates with observed mutation rates in de novo mutation (DNM) data [2] (Figure 1E). We identified sites with <10% or >0.90% methylation level as proxies for unmethylated and methylated sites, respectively. We then compared

the observed DNM rates in different 4mer contexts against our estimated mutation rates for unmethylated and (fully) methylated CpGs. Both comparisons showed strong correlations (Pearson's r=0.82 and 0.97), providing validation of our estimates in a completely independent dataset.

On the other hand, the inter-species comparison provides another type of sanity check and validation. The strong correlation of context-dependent mutation rate estimates between human and rhesus macaque—which stem from completely independent studies and are unlikely to share the same technical artifacts—further reinforces that our findings capture deeply conserved mutation patterns rather than dataset-specific noise.

2- Can we see a model comparison against strong baselines, not just internal AIC comparisons among their own parameterizations. E.g. the 7-mer model from Aggarwala, or also the epigenomically-informed models e.g Roulette?
2b. Ideally, we'd see an application of the improved model, eg to compare how the models improve ability to model constraint in populations. I do appreciate this might be a little bit out of scope for the current paper.

In addition to the comparison with DNMs, we also included a comparison of our model's estimates with three previously published models to validate our results: one study that uses extremely rare variants as proxy for germline mutations without distinguishing between unmethylated and methylated cytosines [3], and two gnomAD mutation models [4,5] that estimate mutation rates across varying methylation bins (3 and 16 bins respectively) (S3 Fig). Our results show high concordance with findings from these existing models while providing more interpretability.

We have clarified the motivation and scope of this study to focus on the biology underlying context-dependent mutation rate variation at CpG sites–specifically the distinct properties of unmethylated and methylated cytosine as mutational substrates–instead of presenting a novel or alternative mutation rate model. As such, we believe comparing model performance with other models or testing whether the model can improve constraint prediction is out of the scope of this study.

3- I would wish for a little bit of mechanistic insight (particularly in light of their modularity claims)? For example: do contexts predicted to be extreme correspond to known DNA-shape features, or have correlation with nucleosome occupancy, replication timing? Strand biases? If they analyzed somatic mutations they could check POLE mutants and MMR mutants.

We appreciate the reviewer's suggestion. To investigate how additional genomic features interact with context or methylation, we analyzed CpG mutation rates stratified by replication direction and replication timing in human, chimpanzee, and rhesus. We find that the rank order of contexts by mutation rate remains consistent regardless of replication direction, although C>T mutation rates are slightly elevated for the leading strand at methylated sites (Figure R1-3A), echoing findings in previous studies [6]. Similarly, late replicating regions have higher mutation rates at CpG sites in all three species (Figure R1-3B), and early replicating regions have lower mutation rates, both consistent with previous studies that have reported that late replicating regions are more mutable [7,8]. Overall, the rank order and relative impact of specific 4-mer contexts remained relatively consistent across all strata of replication direction and timing. These observations lead us to believe that replication is a modifier of absolute CpG mutation rate but not a significant driver or modifier of context effects at CpG sites and have decided not to report it in our manuscript.

Regarding DNA shape, our leading hypothesis for the deep conservation of context effects at unmethylated CpGs (some shared as far distantly as in silkworm and observed even at non-CpG sites) is that they reflect intrinsic DNA structural properties. We compared our mutation rate estimates with DNA shape features of CpG containing sequences predicted by DNAshapeR [9,10] but found no strong correlation between any single parameter across 4-mer contexts. This suggests that the structural influence on mutability likely arises from complex, non-linear combinations of physical features rather than a single dominant factor. Because a definitive structural model requires specialized biophysical simulations, we are currently establishing collaborations with experts in DNA structural biology to pursue this in a dedicated follow-up study.

Finally, the more pronounced differences in context effects at methylated in chimpanzees suggest that certain modifiers likely act downstream of methylation (e.g., deamination or repair). We agree these mechanisms merit deeper investigation, and a mechanistic understanding of these evolutionary shifts is a key objective for our future work. However, given that these factors do not fundamentally alter the context effect estimates reported here, we believe a more detailed characterization is beyond the scope of this manuscript and better suited for a follow-up study.

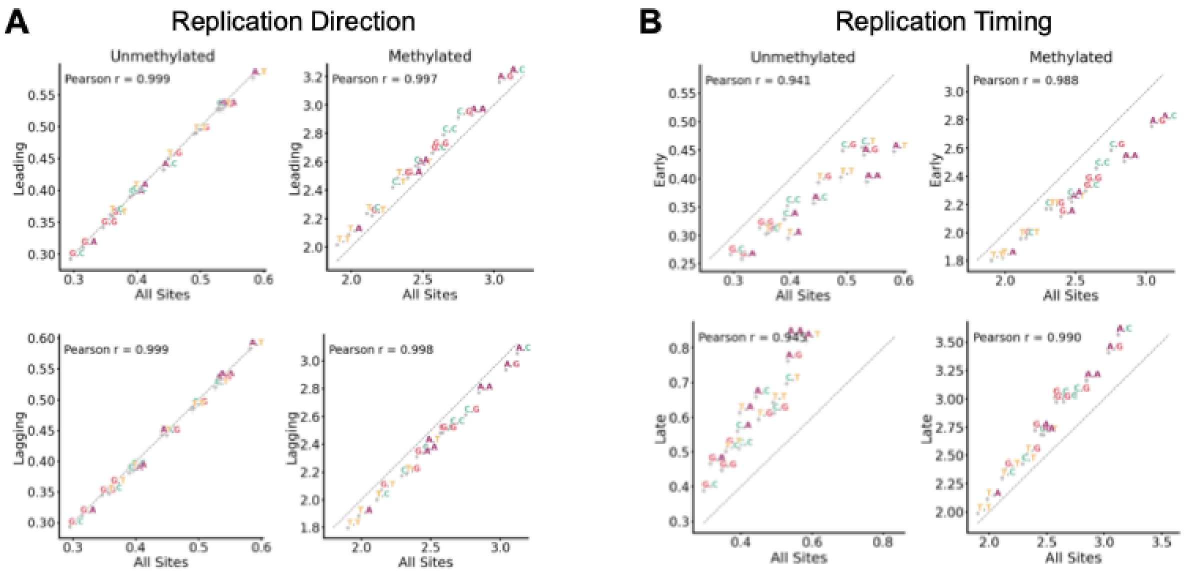

Figure R1. Comparing scaled mutation rate estimates for human across all sites on the x-axis and sites stratified by either A. replication direction with leading strand (top) or lagging strand (bottom) on the y-axis when unmethylated (left) and methylated (right), or B. replication timing with early replication quartile (top) or late replication quartile (bottom) on the y-axis when unmethylated (left) and methylated (right). Dashed line indicates x=y. Points are labeled by the nucleotides upstream and downstream of the CpG, with error bars representing 95% confidence intervals.

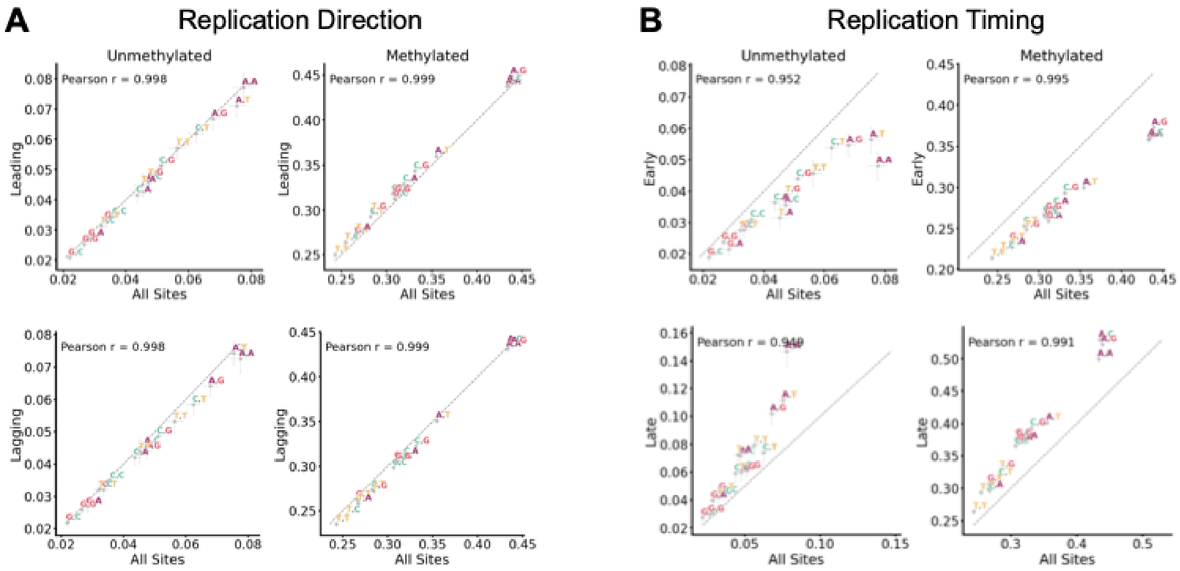

Figure R2. Same as Fig. R1, but for chimpanzee.

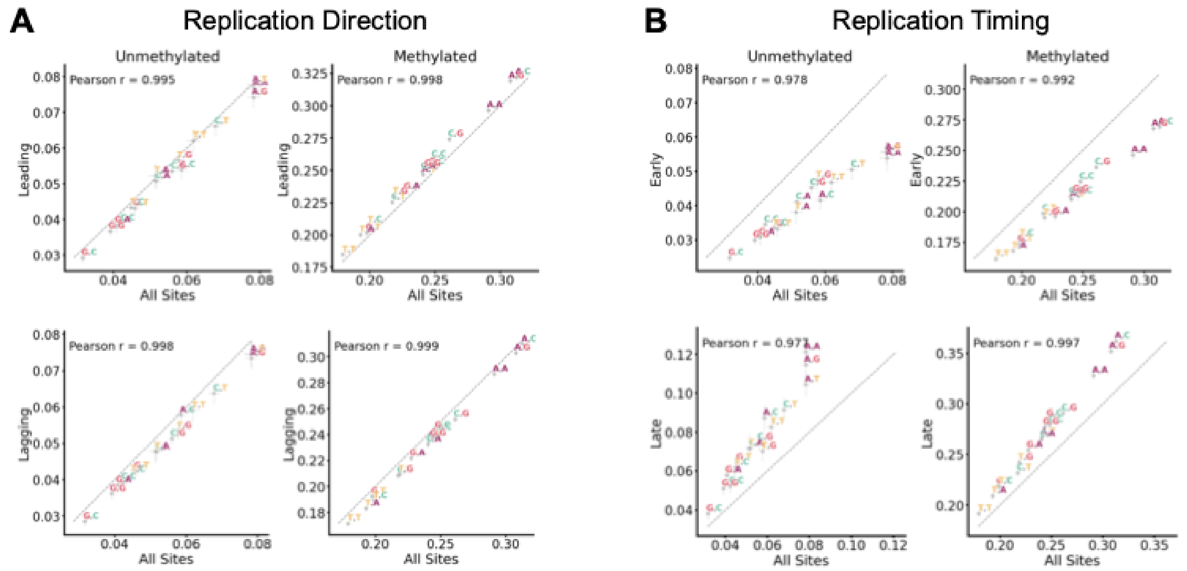

Figure R3. Same as Fig. R1, but for rhesus macaque.

Reviewer #2:

Major Comments

1. Exclusion of singletons and the zero-class approximation

The exclusion of singleton variants violates the standard zero-class Poisson approximation that links mutation rate to presence/absence of SNVs, i.e. P(SNV) = 1 − e^(−μT). As currently implemented, this filtering step alters the relationship between mutation rate and observability in a nontrivial way. I would therefore suggest either (i) including singletons in the analysis, or (ii) explicitly and analytically investigating the consequences of singleton exclusion for the approximation being used.

We agree with the reviewer that excluding singletons can systematically bias our results. Based on this, we have now included singletons in the analysis of all primate species.

The authors appropriately discuss limitations of the Poisson approximation in the Discussion, particularly with respect to variation in coalescent times across sites. It would be useful to extend this discussion to note that residual variance in mutation rate (i.e. variance not captured by the model) could affect the ranking of sequence contexts.

We also thank the reviewer for pointing out the issue of residual variance, and have added a sentence to acknowledge this limitation:

“We also note that residual variance in mutation rate that arise from mis-specification or unmodeled biological determinants in our modeling framework could introduce uncertainty in the inferred ranking of sequence contexts, especially those with similar mutation rates.”

2. Potential role of biased gene conversion in interspecies comparisons

Judging by the effect of sequence context on differences between species, one possible explanation for some of the observed differences is biased gene conversion (BGC). This is especially relevant because cohort sample sizes differ substantially between species, and the average allele frequencies of observed variants are therefore also very different.

While a full investigation of interactions between BGC and CpG mutability may be beyond the scope of the present work, a simple sensitivity analysis would substantially strengthen the interpretation. For example, testing whether differences in mutational spectra are associated with SNV frequency or local recombination rate would help assess the contribution of BGC to the reported interspecies differences.

We fully agree with the reviewer that GC-biased gene conversion has profound influence on the frequency of C>T polymorphisms in a population, thus may bias the relative mutation rates compared to other weak-to-strong or gBGC-neutral mutations. However, the strength of gBGC is not expected to differ substantially across contexts and thus unlikely to lead to significant biases in estimated context effects or ranks of contexts in mutability.

To address this directly with data, we stratified CpG sites by recombination rate into four bins covering roughly the same number of CpG sites: high (> 0.389), mid1 (≤ 0.389), mid2 (≤ 0.026), and low (≤ 4.877e-05). We then applied our model to each recombination bin separately to estimate context-specific mutation rates of methylated and unmethylated CpGs. Consistent with our expectation, we find highly concordant estimates from all sites compared to those of each recombination bins for methylated CpGs (Pearson’s r > 0.99) and high but imperfect correlation for unmethylated CpGs (Pearson’s r>0.97) (S10 Fig). The reduction of correlation at unmethylated sites may be explained by noisier estimation due to smaller numbers of sites with low methylation levels. We now report these results as a supplemental figure (S10 Fig) and include a discussion about this in Methods.

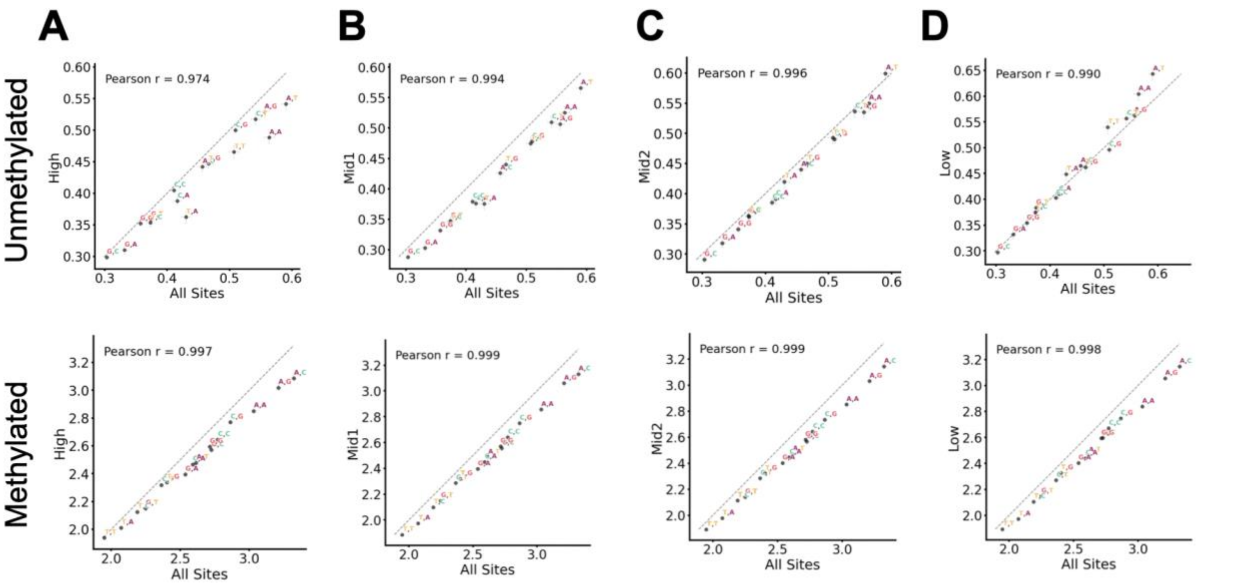

S10 Fig. Context-specific scaled mutation rate estimates are consistent across recombination rate strata. A. Comparison of scaled mutation rate estimates across all sites versus sites stratified by recombination rate, shown separately for unmethylated (top) and methylated (bottom) CpGs. Panels correspond to recombination rate quartiles: **A.** highest (“High”, > 0.389), **B** upper-middle (“Mid1”, ≤ 0.389), **C.** lower-middle (“Mid2”, ≤ 0.026), and **D.** lowest (“Low”, ≤ 4.877×10^{-5}). The dashed line indicates $x = y$. Points are labeled by the upstream and downstream nucleotide flanking the CpG, and error bars denote 95% confidence intervals.

Minor Comments

1. Mechanistic interpretation of CpG mutability

In the Introduction, the authors state: “A widely accepted mechanism underlying the mutagenic effect of methylation is the spontaneous deamination of 5-methylated cytosine (5mC), which occurs at a much higher rate than for unmethylated cytosine [21].”

While deamination of 5mC is certainly an important mechanism, it is less clear that there is consensus that this mechanism alone explains the high mutability of CpG sites. In addition to the replication-related papers cited by the authors, there is extensive literature linking CpG mutation rates to cell division rate and replication-associated processes.

We agree with the reviewer that the high mutability of CpG sites likely arises from a combination of mechanisms beyond spontaneous deamination alone and have accordingly modified this paragraph in the Introduction:

Old text:

However, not all C>T mutations at CpG sites arise from deamination and faulty/inefficient repair. Recent work on human DNA polymerase ε suggests that mis-incorporation of nucleotides during replication can also contribute to

CpG>TpG mutations, as this enzyme shows a bias toward incorporating adenine opposite 5mC and even unmethylated cytosine in a CpG context [12,23].

New text:

However, not all C>T mutations at CpG sites arise from faulty/inefficient repair following deamination; replication-associated errors independent of deamination likely also contribute. For example, analysis of de novo CpG>TpG mutations reveals strong asymmetry with regard to replication direction, showing up to 33% higher mutation rate on the lagging strand [6]. In addition, recent work on human DNA polymerase ε suggests that this enzyme exhibits a bias toward mis-incorporating adenine opposite 5mC and even unmethylated cytosine in a CpG context [11,12].

2. Framing as a mutation rate model

In introduction the authors suggest that their framework could be used directly as a mutation rate model. If this is the intended framing, it would be informative to compare the predictive performance of the proposed model against existing mutation rate models. Alternatively, the authors might consider reframing the study more explicitly as an investigation of the biological determinants of CpG mutability.

We appreciate this suggestion, and have rewritten parts of the Introduction to communicate that this study is intended as an investigation of the biology underlying germline CpG mutation rate variation, and have removed parts that suggest we are presenting an alternative mutation rate model:

Old text:

To evaluate these mechanisms, we need a mutation model that explicitly considers both methylation state and sequence context for each CpG site. Such a model would enable us to disentangle the contribution of a well-defined biochemical mechanism (i.e., 5mC deamination) from additional context effects that may arise from DNA replication, damage, repair, or protein binding.

Here, we introduce a framework for modeling CpG mutation rates across sequence contexts and methylation levels. Unlike existing models that ignore methylation [31,32] or treat it as a categorical variable [3,33], our approach incorporates methylation as a continuous predictor, enabling us to disentangle the contributions of methylation and local sequence context while leveraging information from CpG sites across different methylation levels.

New text:

Because 5mC and unmethylated cytosines possess distinct biochemical properties, quantifying their mutational profiles separately can help distinguish between these competing mechanisms. This necessitates a framework capable of decoupling the influence of methylation from sequence context effects, allowing for the estimation of mutation patterns at both methylated and unmethylated CpGs.

Here, we develop a modeling framework to investigate the biological determinants of CpG mutability by explicitly considering both methylation state and sequence context at each CpG site. We build on existing context-dependent mutation models by incorporating methylation as a continuous predictor, enabling us to disentangle the contributions of methylation and local sequence context while leveraging information from CpG sites across varying methylation levels.

Reviewer #3:

Could part of the reason for the finding that mutation rates of different contexts vary more than methylation can explain be due to the fact that WGBS data cannot distinguish between methylation and hydroxymethylation? If hydroxymethylated sites are labelled as methylated but don't have the increased mutation rates, how does that affect the analysis?

We appreciate the reviewer's concerns about hydroxymethylated sites. To address this, we used published 5hmC calls from TET-assisted bisulfite sequencing in human embryonic stem cells [16] to test how 5hmC levels can obscure or bias estimation of context dependent mutation rates of 5mC. We ran our model on gnomAD polymorphisms after excluding all sites with any recorded 5hmC signal and found that the context-dependent mutation rate estimates were nearly perfectly concordant with those estimated using all sites (S8 Fig). This suggests that hydroxy-methylated sites mis-labeled as methylated sites does not have an appreciable effect on the estimation of context-specific mutation rates at unmethylated cytosine and 5mC. We have included these results as a supplemental figure (S8 Fig) and a discussion in Methods.

Due to the lack of 5hmC data in non-human species, we cannot evaluate the impact of 5hmC on our estimation of mutation rates in chimpanzee and rhesus macaque. Nonetheless, assuming conservation in the CpG hydroxy-methylation

landscape in the germline across primates, mis-measurement of methylation level due to 5hmC is unlikely to lead to any substantial biases.

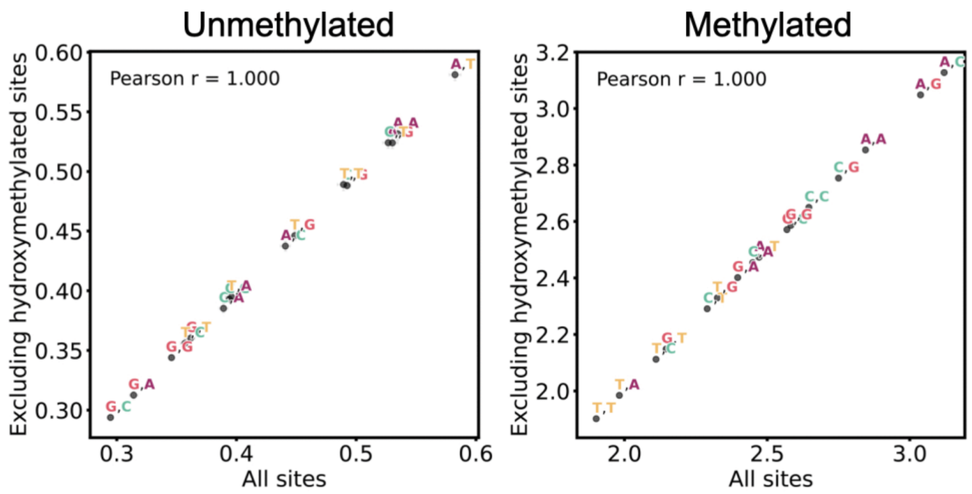

S8 Fig. **Scaled mutation rate estimates are nearly perfectly concordant after excluding hydroxy-methylated (5hmC) sites.** Comparison of the model's scaled mutation rate estimates for unmethylated and methylated sites when using all sites and when excluding sites with a non-zero 5hmC level as measured in human ES-cells. Points are labeled by the upstream and downstream nucleotide context flanking the CpG, and error bars denote 95% confidence intervals around the estimates.

The study removes repeat masked regions but I don't think it masks or in others ways adjusts for differences in the number of covered samples across regions in the polymorphism data. Since GC-content affects both the number of covered samples in a region and the distributions of CpG contexts, it could confound the analysis of how a specific context affects the mutation rate. I think it is worthwhile testing this either by masking regions that have fewer covered samples or by stratifying by GC-content.

We thank the reviewer for noticing this issue. We re-examined the polymorphism datasets for our primate species to determine whether the distribution of callable alleles differed substantially across sequence contexts. Reassuringly, we found no significant differences in sample coverage across contexts in the polymorphism datasets for human, chimpanzee, and rhesus macaque. We have included this finding as a supplemental figure (S9 Fig) and a discussion in Methods.

In addition, the strong consistency of our estimates between independent datasets from human and rhesus macaque further suggests that these context-dependent mutation patterns likely reflect conserved biology rather than technical artifacts related to coverage or GC-content.

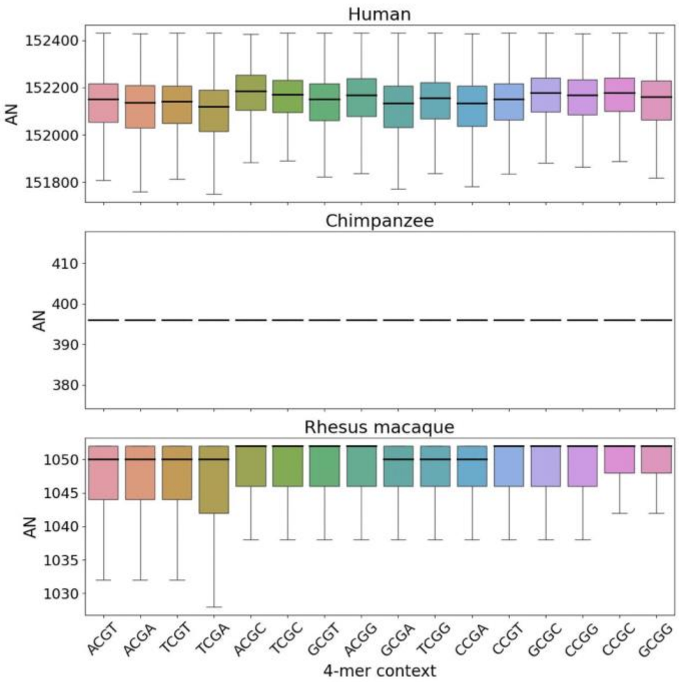

S9 Fig. Callable allele coverage across 4-mer contexts in primate polymorphism datasets. Distribution of the number of callable alleles by 4-mer sequence context in the human, chimpanzee, and rhesus macaque polymorphism dataset. Boxes show the median and interquartile range, with whiskers extending to 1.5x the interquartile range.

References

1. Han S, Riyahi S, Huang X, Kuhlwilm M. A curated dataset of great ape genome diversity. Sci Data. 2025;12:1835. https://doi.org/10.1038/s41597-025-06124-z

2. Palsson G, Hardarson MT, Jonsson H, Steinthorsdottir V, Stefansson OA, Eggertsson HP, et al. Complete human recombination maps. Nature. 2025;639:700–7. https://doi.org/10.1038/s41586-024-08450-5

3. Carlson J, Locke AE, Flickinger M, Zawistowski M, Levy S, Myers RM, et al. Extremely rare variants reveal patterns of germline mutation rate heterogeneity in humans. Nat Commun. 2018;9:3753. https://doi.org/10.1038/s41467-018-05936-5

4. Chen S, Francioli LC, Goodrich JK, Collins RL, Kanai M, Wang Q, et al. A genomic mutational constraint map using variation in 76,156 human genomes. Nature. 2024;625:92–100. https://doi.org/10.1038/s41586-023-06045-0

5. Karczewski KJ, Francioli LC, Tiao G, Cummings BB, Alföldi J, Wang Q, et al. The mutational constraint spectrum quantified from variation in 141,456 humans. Nature. 2020;581:434–43. https://doi.org/10.1038/s41586-020-2308-7

6. Seplyarskiy VB, Sunyaev S. The origin of human mutation in light of genomic data. Nat Rev Genet. 2021;22:672–86. https://doi.org/10.1038/s41576-021-00376-2

7. Koren A, Polak P, Nemesh J, Michaelson JJ, Sebat J, Sunyaev SR, et al. Differential Relationship of DNA Replication Timing to Different Forms of Human Mutation and Variation. Am J Hum Genet. 2012;91:1033–40. https://doi.org/10.1016/j.ajhg.2012.10.018

8. Goldberg ME, Harris K. Mutational Signatures of Replication Timing and Epigenetic Modification Persist through the Global Divergence of Mutation Spectra across the Great Ape Phylogeny. Corbett-Detig R, editor. Genome Biol Evol. 2022;14:evab104. https://doi.org/10.1093/gbe/evab104

9. Rao S, Chiu T-P, Kribelbauer JF, Mann RS, Bussemaker HJ, Rohs R. Systematic prediction of DNA shape changes due to CpG methylation explains epigenetic effects on protein–DNA binding. Epigenetics Chromatin. 2018;11:6. https://doi.org/10.1186/s13072-018-0174-4

10. Chiu T-P, Rao S, Mann RS, Honig B, Rohs R. Genome-wide prediction of minor-groove electrostatic potential enables biophysical modeling of protein–DNA binding. Nucleic Acids Res. 2017;45:12565–76. https://doi.org/10.1093/nar/gkx915

11. Tomkova M, McClellan M, Kriaucionis S, Schuster-Böckler B. DNA Replication and associated repair pathways are involved in the mutagenesis of methylated cytosine. DNA Repair. 2018;62:1–7. https://doi.org/10.1016/j.dnarep.2017.11.005

12. Tomkova M, McClellan MJ, Crevel G, Shahid AM, Mozumdar N, Tomek J, et al. Human DNA polymerase ε is a source of C>T mutations at CpG dinucleotides. Nat Genet. 2024;56:2506–16. https://doi.org/10.1038/s41588-024-01945-x

13. Karczewski KJ, Francioli LC, Tiao G, Cummings BB, Alföldi J, Wang Q, et al. The mutational constraint spectrum quantified from variation in 141,456 humans. Nature. 2020;581:434–43. https://doi.org/10.1038/s41586-020-2308-7

14. Seplyarskiy V, Koch EM, Lee DJ, Lichtman JS, Luan HH, Sunyaev SR. A mutation rate model at the basepair resolution identifies the mutagenic effect of polymerase III transcription. Nat Genet. 2023;55:2235–42. https://doi.org/10.1038/s41588-023-01562-0

15. Adams CJ, Conery M, Auerbach BJ, Jensen ST, Mathieson I, Voight BF. Regularized sequence-context mutational trees capture variation in mutation rates across the human genome. Balding D, editor. PLOS Genet. 2023;19:e1010807. https://doi.org/10.1371/journal.pgen.1010807

16. Yu M, Hon GC, Szulwach KE, Song C-X, Zhang L, Kim A, et al. Base-Resolution Analysis of 5-Hydroxymethylcytosine in the Mammalian Genome. Cell. 2012;149:1368–80. https://doi.org/10.1016/j.cell.2012.04.027
