# Supplementary material for: Sequence context and methylation interact to shape germline mutation rate variation at CpG sites
Source: PLoS Genet. 2026 Jun 1;22(6):e1011957. doi: 10.1371/journal.pgen.1011957 (PMC13245866; doi:10.1371/journal.pgen.1011957)
Supplement: Attachment — Submitted filename: Chandra_Gao_Review.docx [file pgen.1011957.s018.docx]

**Referee Report**

This study by Chandra & Gao presents a detailed investigation of germline mutagenesis at CpG sites, approached from three complementary angles: (1) the effect of methylation, (2) the influence of extended sequence context, and (3) the evolution of CpG mutability. Despite some technical concerns discussed below, the main conclusions of the paper appear defensible, and the overall scope of the work is well aligned with PLOS Genetics.

**Major Comments**

**1. Exclusion of singletons and the zero-class approximation**

The exclusion of singleton variants violates the standard zero-class Poisson approximation that links mutation rate to presence/absence of SNVs, i.e. P(SNV) = 1 − e^(−μT). As currently implemented, this filtering step alters the relationship between mutation rate and observability in a nontrivial way. I would therefore suggest either (i) including singletons in the analysis, or (ii) explicitly and analytically investigating the consequences of singleton exclusion for the approximation being used.

The authors appropriately discuss limitations of the Poisson approximation in the Discussion, particularly with respect to variation in coalescent times across sites. It would be useful to extend this discussion to note that residual variance in mutation rate (i.e. variance not captured by the model) could affect the ranking of sequence contexts.

**2. Potential role of biased gene conversion in interspecies comparisons**

Judging by the effect of sequence context on differences between species, one possible explanation for some of the observed differences is biased gene conversion (BGC). This is especially relevant because cohort sample sizes differ substantially between species, and the average allele frequencies of observed variants are therefore also very different.

While a full investigation of interactions between BGC and CpG mutability may be beyond the scope of the present work, a simple sensitivity analysis would substantially strengthen the interpretation. For example, testing whether differences in mutational spectra are associated with SNV frequency or local recombination rate would help assess the contribution of BGC to the reported interspecies differences.

**Minor Comments**

**1. Mechanistic interpretation of CpG mutability**

In the Introduction, the authors state*: “A widely accepted mechanism underlying the mutagenic effect of methylation is the spontaneous deamination of 5-methylated cytosine (5mC), which occurs at a much higher rate than for unmethylated cytosine [21].”*

While deamination of 5mC is certainly an important mechanism, it is less clear that there is consensus that this mechanism alone explains the high mutability of CpG sites. In addition to the replication-related papers cited by the authors, there is extensive literature linking CpG mutation rates to cell division rate and replication-associated processes.

**2. Framing as a mutation rate model**

In introduction the authors suggest that their framework could be used directly as a mutation rate model. If this is the intended framing, it would be informative to compare the predictive performance of the proposed model against existing mutation rate models. Alternatively, the authors might consider reframing the study more explicitly as an investigation of the biological determinants of CpG mutability.

I am signing my review,
Vladimir Seplyarskiy
