# Supplementary material for: Sequence context and methylation interact to shape germline mutation rate variation at CpG sites
Source: PLoS Genet. 2026 Jun 1;22(6):e1011957. doi: 10.1371/journal.pgen.1011957 (PMC13245866; doi:10.1371/journal.pgen.1011957)
Supplement: Peer review history

# Peer review: Decision Letter 0

**DOI:** 10.1371/journal.pgen.1011957.r001

**Authors:** David Balding, Kelly A. Dyer

10 Feb 2026

PGENETICS-D-25-01230

Sequence context and methylation interact to shape germline mutation rate variation at CpG sites

PLOS Genetics

Dear Dr. Gao,

Thank you for submitting your manuscript to PLOS Genetics. After careful consideration, we feel that it has merit but does not fully meet PLOS Genetics's publication criteria as it currently stands. Therefore, we invite you to submit a revised version of the manuscript that addresses the points raised during the review process.

Please submit your revised manuscript by Apr 11 2026 11:59PM. If you will need more time than this to complete your revisions, please reply to this message or contact the journal office at plosgenetics@plos.org. When you're ready to submit your revision, log on to https://www.editorialmanager.com/pgenetics/ and select the 'Submissions Needing Revision' folder to locate your manuscript file.

Please include the following items when submitting your revised manuscript:

* A letter that responds to each point raised by the editor and reviewer(s). You should upload this letter as a separate file labeled 'Response to Reviewers'. This file does not need to include responses to any formatting updates and technical items listed in the 'Journal Requirements' section below.

* A marked-up copy of your manuscript that highlights changes made to the original version. You should upload this as a separate file labeled 'Revised Manuscript with Track Changes'.

* An unmarked version of your revised paper without tracked changes. You should upload this as a separate file labeled 'Manuscript'.

If you would like to make changes to your financial disclosure, competing interests statement, or data availability statement, please make these updates within the submission form at the time of resubmission. Guidelines for resubmitting your figure files are available below the reviewer comments at the end of this letter.

We look forward to receiving your revised manuscript.

Kind regards,

David Balding

Academic Editor

PLOS Genetics

Kelly Dyer

Section Editor

PLOS Genetics

Aimée Dudley

Editor-in-Chief

PLOS Genetics

Anne Goriely

Editor-in-Chief

PLOS Genetics

**Additional Editor Comments:**

Just two minor comments to add to the reviewer comments:

Fig 1A is too dense to be of much use. Connecting line segments are a bit misleading when bins are modelled independently, they can be a visual aid but here the overlapping undermines that. I can't at the moment recommend a better way to present the data - it will require some exploration.

Table 1: since you discuss number of parameters in each model perhaps this should be included in the table. Your own discussion in the table caption illustrates that AIC is not much use when sample sizes are large, because model complexity is insufficiantly penalised. BIC may be more useful.

**Journal Requirements:**

1) We ask that a manuscript source file is provided at Revision. Please upload your manuscript file as a .doc, .docx, .rtf or .tex. If you are providing a .tex file, please upload it under the item type u2018LaTeX Source Fileu2019 and leave your .pdf version as the item type u2018Manuscriptu2019.

2) Please upload all main figures as separate Figure files in .tif or .eps format. For more information about how to convert and format your figure files please see our guidelines:

https://journals.plos.org/plosgenetics/s/figures

**Reviewers' comments:**

Reviewer's Responses to Questions

**Comments to the Authors:**

Reviewer #1: In this study, the authors report that germline mutation rates at CpG dinucleotides are affected by the broader context in a manner dependant on the methylation of the C. This report of statistical interaction of the DNA methylation and the oligonucleotide mutation risk is to my knowledge novel. The study is however not fully developed in terms of validating the effect in independant data, comparing against baselines, and showing at least some mechanistic implications of the result.

1- Could they show predictive value on truly independent mutation data, ideally de novo germline mutations (trios) rather than polymorphisms; alternatively or in addition, somatic mutation analysis would certainly not be uninteresting either.

This should support the claim that their methylation * context model improves prediction beyond existing sequence-context-only models and beyond methylation alone.

2- Can we see a model comparison against strong baselines, not just internal AIC comparisons among their own parameterizations. E.g. the 7-mer model from Aggarwala, or also the epigenomically-informed models e.g Roulette?

2b. Ideally, we'd see an application of the improved model, eg to compare how the models improve ability to model constraint in populations. I do appreciate this might be a little bit out of scope for the current paper.

3- I would wish for a little bit of mechanistic insight (particularly in light of their modularity claims)? For example: do contexts predicted to be extreme correspond to known DNA-shape features, or have correlation with nucleosome occupancy, replication timing? Strand biases? If they analyzed somatic mutations they could check POLE mutants and MMR mutants.

Reviewer #2: Should be attached

Reviewer #3: The authors estimate mutation rates for methylated and unmethylated CpGs in different

sequence contexts using human genetic variation data using a new regression strategy that accounts for recurrent mutations. They find that mutation rates across CpG contexts vary more than methylation alone can explain. They also extend the analysis to other species and see both conserved and species-specific patterns.

The study uses a new regression strategy to draw inferences about the mutation processes at CpG sites and offers valuable insights. I think it is worth publishing, and I only have two issues/questions I think should be addressed.

1) Could part of the reason for the finding that mutation rates of different contexts vary more than methylation can explain be due to the fact that WGBS data cannot distinguish between methylation and hydroxymethylation? If hydroxymethylated sites are labelled as methylated but don’t have the increased mutation rates, how does that affect the analysis?

2) The study removes repeatmasked regions but I don’t think it masks or in others ways adjusts for differences in the number of covered samples across regions in the polymorphism data. Since GC-content affects both the number of covered samples in a region and the distributions of CpG contexts, it could confound the analysis of how a specific context affects the mutation rate. I think it is worthwhile testing this either by masking regions that have fewer covered samples or by stratifying by GC-content.

**********

**Have all data underlying the figures and results presented in the manuscript been provided?**

Large-scale datasets should be made available via a public repository as described in the *PLOS Genetics* data availability policy, and numerical data that underlies graphs or summary statistics should be provided in spreadsheet form as supporting information.

Reviewer #1: Yes

Reviewer #2: Yes

Reviewer #3: **No:**

**********

PLOS authors have the option to publish the peer review history of their article (what does this mean?). If published, this will include your full peer review and any attached files.

If you choose “no”, your identity will remain anonymous but your review may still be made public.

**Do you want your identity to be public for this peer review?** For information about this choice, including consent withdrawal, please see our Privacy Policy.

Reviewer #1: No

Reviewer #2: **Yes:**Vladimir Seplyarskiy

Reviewer #3: **Yes:**Søren Besenbacher

[NOTE: If reviewer comments were submitted as an attachment file, they will be attached to this email and accessible via the submission site. Please log into your account, locate the manuscript record, and check for the action link "View Attachments". If this link does not appear, there are no attachment files.]

**Figure resubmission:**

While revising your submission, we strongly recommend that you use PLOS’s NAAS tool (https://ngplosjournals.pagemajik.ai/artanalysis) to test your figure files. NAAS can convert your figure files to the TIFF file type and meet basic requirements (such as print size, resolution), or provide you with a report on issues that do not meet our requirements and that NAAS cannot fix.

After uploading your figures to PLOS’s NAAS tool - https://ngplosjournals.pagemajik.ai/artanalysis, NAAS will process the files provided and display the results in the "Uploaded Files" section of the page as the processing is complete. If the uploaded figures meet our requirements (or NAAS is able to fix the files to meet our requirements), the figure will be marked as "fixed" above. If NAAS is unable to fix the files, a red "failed" label will appear above. When NAAS has confirmed that the figure files meet our requirements, please download the file via the download option, and include these NAAS processed figure files when submitting your revised manuscript.

**Reproducibility:**

To enhance the reproducibility of your results, we recommend that authors of applicable studies deposit laboratory protocols in protocols.io, where a protocol can be assigned its own identifier (DOI) such that it can be cited independently in the future. Additionally, PLOS ONE offers an option to publish peer-reviewed clinical study protocols. Read more information on sharing protocols at https://plos.org/protocols?utm_medium=editorial-email&utm_source=authorletters&utm_campaign=protocols

Attachment Submitted filename: Chandra_Gao_Review.docx

# Author comment: Author response to Decision Letter 1

**DOI:** 10.1371/journal.pgen.1011957.r002

10 Apr 2026

Attachment Submitted filename: Response_to_reviewers_v2.pdf

# Peer review: Decision Letter 1

**DOI:** 10.1371/journal.pgen.1011957.r003

**Authors:** David Balding, Kelly A. Dyer

5 May 2026

Dear Dr Gao,

We are pleased to inform you that your manuscript entitled "Sequence context and methylation interact to shape germline mutation rate variation at CpG sites" has been editorially accepted for publication in PLOS Genetics. Congratulations!

One reviewer has suggested minor revision which the authors should consider responding to in preparing the final version. Any changes to the text should be minor and any additional table/figure should be provided as supplementary information, not in the main paper.

Before your submission can be formally accepted and sent to production you will need to complete our formatting changes, which you will receive in a follow up email. Please be aware that it may take several days for you to receive this email; during this time no action is required by you. Please note: the accept date on your published article will reflect the date of this provisional acceptance, but your manuscript will not be scheduled for publication until the required changes have been made.

Once your paper is formally accepted, an uncorrected proof of your manuscript will be published online ahead of the final version, unless you’ve already opted out via the online submission form. If, for any reason, you do not want an earlier version of your manuscript published online or are unsure if you have already indicated as such, please let the journal staff know immediately at plosgenetics@plos.org.

In the meantime, please log into Editorial Manager at https://www.editorialmanager.com/pgenetics/, click the "Update My Information" link at the top of the page, and update your user information to ensure an efficient production and billing process. Note that PLOS requires an ORCID iD for all corresponding authors. Therefore, please ensure that you have an ORCID iD and that it is validated in Editorial Manager. To do this, go to ‘Update my Information’ (in the upper left-hand corner of the main menu), and click on the Fetch/Validate link next to the ORCID field.  This will take you to the ORCID site and allow you to create a new iD or authenticate a pre-existing iD in Editorial Manager.

If you have a press-related query, or would like to know about making your underlying data available (as you will be aware, this is required for publication), please see the end of this email. If your institution or institutions have a press office, please notify them about your upcoming article at this point, to enable them to help maximise its impact. Inform journal staff as soon as possible if you are preparing a press release for your article and need a publication date.

Thank you again for supporting open-access publishing; we are looking forward to publishing your work in PLOS Genetics!

Yours sincerely,

David Balding

Academic Editor

PLOS Genetics

Kelly Dyer

Section Editor

PLOS Genetics

Aimée Dudley

Editor-in-Chief

PLOS Genetics

Anne Goriely

Editor-in-Chief

PLOS Genetics

www.plosgenetics.org

BlueSky: @plos.bsky.social

----------------------------------------------------

Reviewer's **Comments to the Authors:**

Reviewer #1: I thank the authors for the additional analysis, which appears satisfactory. As a minor comment I do wish they included in the paper a figure or table with the result of their analysis of de novo mutations (which was supportive); I could not spot this figure nor it appears mentioned in the text. Similarly so, there is not a reason to not mention the replication time/strand analysis that they have already performed. Overall, congratulations on this solid, focused study reporting an interesting new finding about interplay of DNA methylation and local context.

Reviewer #2: I am satisfied with the responses and believe that manuscript is ready for publication

Reviewer #3: The authors have adequately tested the concerns I raised.

**********

**Have all data underlying the figures and results presented in the manuscript been provided?**

Large-scale datasets should be made available via a public repository as described in the *PLOS Genetics* data availability policy, and numerical data that underlies graphs or summary statistics should be provided in spreadsheet form as supporting information.

Reviewer #1: Yes

Reviewer #2: Yes

Reviewer #3: None

**********

PLOS authors have the option to publish the peer review history of their article (what does this mean?). If published, this will include your full peer review and any attached files.

If you choose “no”, your identity will remain anonymous but your review may still be made public.

**Do you want your identity to be public for this peer review?** For information about this choice, including consent withdrawal, please see our Privacy Policy.

Reviewer #1: No

Reviewer #2: **Yes:**Vladimir Seplyarskiy

Reviewer #3: **Yes:**Søren Besenbacher

----------------------------------------------------

**Data Deposition**

If you have submitted a Research Article or Front Matter that has associated data that are not suitable for deposition in a subject-specific public repository (such as GenBank or ArrayExpress), one way to make that data available is to deposit it in the Dryad Digital Repository. As you may recall, we ask all authors to agree to make data available; this is one way to achieve that. A full list of recommended repositories can be found on our website.

The following link will take you to the Dryad record for your article, so you won't have to re‐enter its bibliographic information, and can upload your files directly:

http://datadryad.org/submit?journalID=pgenetics&manu=PGENETICS-D-25-01230R1

More information about depositing data in Dryad is available at http://www.datadryad.org/depositing. If you experience any difficulties in submitting your data, please contact help@datadryad.org for support.

Additionally, please be aware that our data availability policy requires that all numerical data underlying display items are included with the submission, and you will need to provide this before we can formally accept your manuscript, if not already present.

----------------------------------------------------

**Press Queries**

If you or your institution will be preparing press materials for this manuscript, or if you need to know your paper's publication date for media purposes, please inform the journal staff as soon as possible so that your submission can be scheduled accordingly. Your manuscript will remain under a strict press embargo until the publication date and time. This means an early version of your manuscript will not be published ahead of your final version. PLOS Genetics may also choose to issue a press release for your article. If there's anything the journal should know or you'd like more information, please get in touch via plosgenetics@plos.org.

# Editor report: Acceptance letter

**DOI:** 10.1371/journal.pgen.1011957.r004

**Authors:** David Balding, Kelly A. Dyer

PGENETICS-D-25-01230R1

Sequence context and methylation interact to shape germline mutation rate variation at CpG sites

Dear Dr Gao,

We are pleased to inform you that your manuscript entitled "Sequence context and methylation interact to shape germline mutation rate variation at CpG sites" has been formally accepted for publication in PLOS Genetics! Your manuscript is now with our production department and you will be notified of the publication date in due course.

The corresponding author will soon be receiving a typeset proof for review, to ensure errors have not been introduced during production. Please review the PDF proof of your manuscript carefully, as this is the last chance to correct any errors. Please note that major changes, or those which affect the scientific understanding of the work, will likely cause delays to the publication date of your manuscript.

Soon after your final files are uploaded, unless you have opted out or your manuscript is a front-matter piece, the early version of your manuscript will be published online. The date of the early version will be your article's publication date. The final article will be published to the same URL, and all versions of the paper will be accessible to readers.

For Research Articles, you will receive an invoice from PLOS for your publication fee after your manuscript has reached the completed accept phase. If you receive an email requesting payment before acceptance or for any other service, this may be a phishing scheme. Learn how to identify phishing emails and protect your accounts at https://explore.plos.org/phishing.

Thank you again for supporting PLOS Genetics and open-access publishing. We are looking forward to publishing your work!

With kind regards,

Anitha Samidurai

PLOS Genetics

On behalf of:

The PLOS Genetics Team

Carlyle House, Carlyle Road, Cambridge CB4 3DN | United Kingdom

plosgenetics@plos.org | +44 (0) 1223-442823

plosgenetics.org | Twitter: @PLOSGenetics
